# *Streptococcus pneumoniae*-associated hemolytic uremic syndrome

**DOI:** 10.1007/s00467-007-0518-y

**Published:** 2008-11-01

**Authors:** Lawrence Copelovitch, Bernard S. Kaplan

**Affiliations:** Department of Pediatrics, Division of Nephrology, The Children’s Hospital of Philadelphia, 34th Street and Civic Center Boulevard, Philadelphia, PA 19104 USA

**Keywords:** *Streptococcus pneumoniae*, Pneumococcal, Hemolytic uremic syndrome, Haemolytic uremic syndrome, Pneumonia, Meningitis

## Abstract

*Streptococcus pneumonia*-associated hemolytic uremic syndrome (HUS) (pneumococcal HUS) is an uncommon condition mainly observed in young children. Early recognition is critical, because of the potential to improve morbidity and mortality. In our review we summarize the pathophysiology, clinical features, diagnostic difficulties and management of this potentially under-diagnosed condition.

## Introduction

Hemolytic uremic syndrome (HUS) in children is usually caused by Shiga-like toxin-producing *E. coli* (STEC). However, HUS is also a complication of invasive pneumococcal infection. The purpose of this review is to describe, in detail, the clinico-pathological entity known as *Streptococcus pneumoniae*-associated HUS (pneumococcal HUS). It is important to discuss this condition separately from other causes of HUS, because, in our experience, cases continue to go unrecognized. We address the pitfalls that arise when trying to make the diagnosis. We highlight the importance of practicing a conservative approach to the use of blood and plasma products in these often critically ill patients. We also show that the acute mortality rate is highest in patients with *S. pneumoniae* meningitis complicated by HUS.

## Historical perspective

Acute renal injury in the form of nephritis has been reported in association with pneumococcal disease since 1872 [1, 2]. Councilman, in 1897 [3], noted that nephritis occurred in 11 out of 102 cases of pneumonia; all the patients were very young children. Schenk et al., in 1970 [4], described three cases of pneumococcal septicemia associated with thrombocytopenia and glomerular and arteriolar thrombosis without mentioning hemolytic anemia or HUS. In 1971 Fischer et al. [5] described the association of *S. pneumoniae* infection with HUS, and, in 1977, Klein et al. [6] first reported this condition in the English literature. Early reports indicated that this rare condition was associated with a very poor clinical outcome. Of the 12 patients reported in the English language literature between 1977 and 1987, 50% died and 67% of the survivors developed chronic kidney disease or hypertension [6–13].

## Incidence and prevalence

Pneumococcal HUS is an uncommon condition and involves 5% of all cases of HUS in children but 38–43% of HUS cases not caused by STEC [14, 15]. The incidence of HUS following invasive pneumococcal infections is estimated at 0.4–0.6% [16, 17]. Many investigators have noted that the incidence of pneumococcal HUS may be substantially higher and is currently underestimated due to a lack of awareness [14, 17–19]. The prevalence of pneumococcal HUS is highest among children under 2 years of age and mirrors the overall incidence of invasive pneumococcal infection. In contrast, this association is extremely uncommon in adults [20, 21]. Of the 85 cases of pneumococcal HUS reported in the English literature [6–14, 16, 18, 19, 22–38], 72% were associated with pneumonia and/or empyema, 29% with meningitis, and 5% with pneumonia and meningitis. Spontaneous bacteremia, pericarditis, peritonitis, and mastoiditis were noted infrequently.

## Pathophysiology

The precise pathophysiology of pneumococcal HUS has not been determined. However, there is evidence of a role for the Thomsen–Friedenreich (TF) cryptantigen [39]. This antigen is a component of the surface structure of erythrocytes, platelets and glomerular endothelial cells and is normally hidden by neuraminic acid. Neuraminidase, produced by pneumococci, cleaves the *n*-acetyl neuraminic acid from the cell surface and exposes the TF antigen. Pre-formed host IgM antibodies then bind the TF antigen and are postulated to initiate the cascade of events leading to HUS. The activated TF antigen is also present on hepatocytes, and this may explain the occurrence of transient hepatic dysfunction in some patients [26, 32].

All serotypes of *S. pneumoniae* can have neuraminidase activity capable of unmasking the TF antigen [22]. Therefore, any invasive pneumococcal infection may result in a range of abnormalities, ranging from isolated hemolytic anemia to full-blown HUS. Serotypes 14, 6B, 9V, 19, 3, 8, 23F [32] and 19A (unpublished observation) are all associated with HUS. Theoretically, different serotypes may produce varying amounts and activities of neuraminidase, thereby influencing the likelihood of a patient’s developing HUS. Some authors have suggested that a heavy bacterial load increases the individual’s risk of developing pneumococcal HUS [31]. Evidence for this hypothesis is based on the relatively high frequency of patients with empyema-associated pneumonia and the rarity of patients who present with spontaneous bacteremia or minor pneumococcal infections. Parenthetically, several other neuraminidase-producing organisms, including *Capnocytophaga canimorsus* [40, 41] and, possibly, influenza A virus, are also reported as causes of HUS [42].

## Clinical features

Features of pneumococcal HUS usually develop 3 to 13 days (most at 7 to 9 days) after the onset of pneumococcus-related symptoms [19, 38]. This chronology is similar to that described for Shiga-like toxin-producing *E. coli*-associated HUS (STEC HUS), in which diarrheal illness typically occurs 7 days (range 1–14 days) prior to the onset of HUS [43]. There is no gender predisposition [19]. Children with pneumococcal HUS are younger at presentation and have more severe initial hospital courses than those with STEC HUS [31]. They also have longer periods of oligo-anuria, more frequent need for acute dialysis (75% vs 59%), longer hospital stays (33 days vs 16 days), more red blood cell and platelet transfusions, and a longer duration of thrombocytopenia [31]. In our review of the 73 patients reported from 1987 to 2007 [14, 16, 18, 19, 22–38] 10.1% developed end-stage renal failure (ESRF) and 12.3% died in the acute phase. These rates are two-to-three-times higher than those of STEC HUS [43, 44, 45]. However, the acute mortality rate of pneumococcal HUS not associated with meningitis is comparable to that of STEC HUS. In contrast, the higher rates of ESRF and chronic kidney disease do not appear to be related to the site of *S. pneumoniae* infection.

The Canadian Paediatric Surveillance Program divided patients with invasive pneumococcal infection and features of HUS into two categories [46]. *Definite* cases were defined by the presence of thrombotic microangiopathy on renal biopsy or autopsy. Some authors have modified this strict definition and consider a positive result of the Coombs’ test (see later) as evidence of a definitive case in the absence of a tissue diagnosis [34]. *Possible* cases were defined as pneumococcal HUS that could not be differentiated from pneumococcal sepsis or disseminated intravascular coagulation (DIC) with secondary organ failure. Other authors [32] have recommended upgrading all *possible* cases to *probable* status, so that the diagnosis is not overlooked.

Whenever there is evidence of renal injury in the context of pneumococcal infection it is important to maintain a broad diagnostic perspective. Acute tubular necrosis occurs in patients with septic shock and DIC. Immune-complex-mediated acute glomerulonephritis is a rare complication of pneumococcal infection [1]. In our experience the major reasons why the diagnosis of pneumococcal HUS may be overlooked are a lack of familiarity with this rare condition, a misdiagnosis of DIC, the coexistence of HUS and DIC, and cases of pneumococcus-associated microangiopathic hemolytic anemia with only mild renal involvement. Another possible explanation is the unavailability of a highly specific laboratory test.

HUS and DIC may be difficult to differentiate from each other because both may involve microangiopathic hemolytic anemia, thrombocytopenia, and renal injury or failure. In most cases of HUS, however, fibrinogen levels, and prothrombin and partial thromboplastin times are normal or slightly elevated, and there is no active bleeding. Unfortunately, when these two conditions occur in the same patient, a diagnosis of HUS may be difficult to make with certainty. Evidence of a thrombotic microangiopathy on renal biopsy can be helpful, but the clinical instability of many patients precludes this procedure. Several investigators have proposed that patients who satisfy the criteria for both HUS and DIC in the setting of pneumococcal infection should be considered as probably having HUS and should be treated accordingly [29, 32].

Early reports suggested that only bacterial isolates from patients with pneumococcal HUS had detectable neuraminidase activity, while pneumococcal isolates of the same serotype cultured from non-HUS patients had no neuramindase activity [11]. These studies erroneously concluded that detection of neuraminidase activity was a highly specific diagnostic tool for pneumococcal HUS. Thomsen–Friedenreich (TF) antigen exposure, an indirect measure of neuraminidase activity, can be assessed by the peanut (*Arachis hypogaea*) lectin-agglutination method. The fluorescin-labeled peanut agglutinin has a high affinity for the normally unexposed TF antigen. Peanut lectin activity was detected in 36 patients with invasive pneumococcal disease [47]. In this study positive test results were obtained in 100% (13/13) of patients with pneumococcal HUS, in 67% (6/9) of patients with pneumococcus-associated hemolytic anemia, and in 43% (6/14) of patients with uncomplicated invasive pneumococcal disease. These results suggest that, in the setting of invasive pneumococcal infection, detectable neuraminidase activity is highly sensitive (100%), but not specific (48%), for pneumococcal HUS. This implies that neuraminidase activity is required but not sufficient to initiate HUS and that other, as yet unknown, host or environmental factors must be present.

The exposed TF antigen results in a variety of antigen–antibody interactions, many of which occur on the plasma membrane of red blood cells. The direct Coombs’ test detects antibodies that coat these surfaces and gives positive results in approximately 90% of cases of pneumococcal HUS [39, 48]. In the presence of invasive pneumococcal infection, difficulties in ABO cross-matching or a positive minor cross-match are useful tools in making the diagnosis. Unfortunately, there are no data regarding the incidence of Coombs’ test positivity in non-HUS forms of pneumococcal infection. While a positive Coombs’ test result is highly sensitive for pneumococcal HUS, the degree of specificity is still unclear.

## Renal pathology

The histological findings in pneumococcal HUS are similar to those in STEC HUS. Fibrin thrombi are seen in the glomerular capillaries in both forms of HUS. Renal cortical necrosis may be present [6]. Occasionally, the fibrin thrombi can extend beyond the capillaries into the afferent arterioles [6]. Varying degrees of mesangial proliferation, focal or widespread glomerulonephritis, crescent formation, and tubular atrophy may occur [6, 11, 12]. The arterial vessels usually appear normal [12]. Immunofluorescence staining is negative except for weak staining for IgM [6, 11, 12]. Special staining with immunofluorescence-labeled peanut agglutinin has been found to be positive in the glomeruli [6, 11, 13, 22] and tubules [12] of several patients. This test is not widely available. These findings support the role of the TF antigen–IgM antibody interaction in the pathogenesis of pneumococcal HUS [12].

## Management

The management of pneumococcal HUS is supportive. Controlling the abnormalities caused by the renal failure, anemia, and thrombocytopenia is essential. In addition, dialysis or hemodiafiltration is often required to maintain electrolyte and fluid balances and adequate nutrition in oligo-anuric patients. Because of the significant incidence of antibiotic resistance in the community, the American Academy of Pediatrics recommends empirically treating critically ill children with possible or proven invasive pneumococcal infections with both vancomycin and an extended-spectrum cephalosporin [35, 49]. Theoretically, because anti-TF IgM antibodies are integral to the pathogenesis, additional pre-formed antibody administration via fresh frozen plasma or unwashed blood products should be avoided. Fresh frozen plasma should be avoided unless there is active bleeding. Furthermore, it is preferable to transfuse washed red blood cells or platelets. More than 95% of the plasma can be effectively removed from blood products by dextran-washing [19]. When hemodiafiltration is performed in young children it is important to avoid priming the dialysis system with unwashed blood products. There is no evidence that plasmapheresis is of value. Several authors have attributed the recent decrease in mortality rate to earlier recognition and a more conservative approach to transfusion with washed blood products [25, 33]. It remains to be seen whether the routine administration of the seven-valent pneumococcal vaccine to infants has effectively decreased the incidence of pneumococcal HUS.

## Outcomes

Since 1987, 73 children [14, 16, 18, 19, 22–38] with pneumococcal HUS have been reported in the English language literature. Of these, 12.3% (9/73) died; 10.1% (7/69) progressed to ESRF; and 16% (11/69) survived, with chronic kidney disease or hypertension. Detailed clinical [33] and follow-up data [34] were unavailable for four patients. The improved mortality rate in the past 20 years may have been the result of the advances in critical care medicine as opposed to changes in disease virulence [31]. A careful analysis of the 69 children with comprehensive clinical data [14, 16, 18, 19, 22–32, 34–38] reveals that only 28% of the patients presented with meningitis. However, 88% of the deaths occurred in this subgroup (clinical data available on 8/9 patient deaths). The estimated mortality rate of pneumococcal meningitis in children without HUS is 8–10% [50], while the mortality rate in patients with pneumococcal meningitis complicated by HUS is 37% (7/19) [16, 22, 23, 26, 29–31, 34]. In marked contrast, pneumococcal HUS not associated with meningitis has a mortality rate of only 2% [14, 16, 18, 19, 22, 24–32, 34–38].

The two largest case series published to date support the notion that the acute mortality rate is largely dependant on the site of infection. In 2002 Brandt et al. [31] described 12 patients: nine had isolated pneumonia and three had pneumococcal HUS with meningitis. There were no deaths in either group. Two of the seven patients with long-term follow-up developed ESRF, and five had normal renal function. In 2001, Nathanson and Deschenes [29] reported their observations on 11 patients: eight had meningitis and three had pneumococcal HUS with an isolated pneumonia. There were no deaths in patients with pneumonia; two developed mild chronic kidney disease, and one recovered completely. Four of the eight patients with meningitis died, three progressed to ESRF, and one recovered completely. The poor prognosis seen in patients with pneumococcal meningitis complicated by HUS is consistent with the earlier observation that the severity of pneumococcal meningitis is related to neuraminidase activity and TF exposure in the central nervous system [51].

## Conclusion

Pneumococcal HUS is a well-characterized condition that may still be under-recognized. The absence of a consistent case definition and the lack of a specific laboratory test may play a role in under-diagnosis and late detection. The potential to improve morbidity and mortality, by the avoidance of plasma infusion or exchange and transfusion of unwashed blood products, makes early recognition important. From a practical point of view, pneumococcal HUS should be suspected when one or more of the following occurs in the context of HUS: a toxic patient, pneumonia, meningitis or other evidence of invasive infection, a hemolytic anemia without reticulocyte response, a positive Coombs’ test result or a positive minor cross-match.

## **Questions** (Answers appear following the reference list)

1. **Which of the following molecules is involved in the pathogenesis of pneumococcal HUS?**
  A. Globotriaosylceramide (Gb3)
  B. von Willebrand factor cleaving protease ADAMTS13
  C. Neuraminic acid
  D. Factor H
  E. Membrane co-factor protein (CD46)
2. **Which of the following laboratory findings is*****NOT*****associated with pneumococcal HUS?**
  A. Microangiopathic hemolytic anemia
  B. Positive result of a Coombs’ test
  C. Positive result of a peanut lectin activity test
  D. Hypocomplementemia
  E. Normal or slightly elevated prothrombin time
3. **Which of the following is associated with a poor outcome in pneumococcal HUS?**
  A. Patient age
  B. Serotype of *S. pneumoniae*
  C. Meningitis
  D. Severity of anemia
  E. All of the above
4. **In the treatment of a patient with pneumococcal HUS all of the following should be avoided*****EXCEPT?***
  A. Continuous venovenous hemofiltration (CVVH)
  B. Transfusion of unwashed packed red blood cells (PRBCs)
  C. Plasmapheresis
  D. Fresh frozen plasma
  E. Transfusion of unwashed platelets
5. **In the comparison of children with pneumococcal HUS and STEC HUS, which of the following is*****FALSE?***
  A. Children with pneumococcal HUS are more likely to require acute dialysis
  B. There is no gender predisposition in either form of HUS
  C. Children with pneumococcal HUS are likely to be older at presentation
  D. Children with pneumococcal HUS are more likely to develop chronic kidney disease
  E. Children with pneumococcal HUS are more likely to require red blood cell and platelet transfusions

## References

[1] Gray BM (2001). Is pneumococcal hemolytic-uremic syndrome a new disease?. Infect Med.

[2] Rake G (1933). Nephritis in pneumococcal infections. Guys Hosp Rep.

[3] Councilman WT (1897). An anatomical and bacteriologic study of acute diffuse nephritis. Am J Med Sci.

[4] Schenk EA, Panke TW, Cole HA (1970). Glomerular and arteriolar thrombosis in pneumococcal septicemia. Arch Pathol.

[5] Fischer K, Poschmann A, Oster H (1971). Hamolyse bei schwerer Pneumonie infolge Neuraminidasewirkung. Monatsschr Kinderheilkd.

[6] Klein PJ, Bulla M, Newman RA, Muller P, Uhlenbruck G, Schaefer HE, Kruger G, Fisher R (1977). Thomsen-Friedenreich antigen in haemolyitc-uraemic syndrome. Lancet.

[7] Moorthy B, Makker SP (1979). Hemolytic-uremic syndrome associated with pneumococcal sepsis. J Pediatr.

[8] Yahav J, Aladjem M, Boichis H, Barzilay Z (1980). Hemolytic uremic syndrome due to pneumococcal sepsis. Int J Pediatr Nephrol.

[9] Seger R, Joller P, Baerlocher K, Kenny A, Dulake C, Leuman E, Spierig M, Hitzig WH (1980). Haemolytic-uremic syndrome associated with neuraminidase-producing microorganisms: treatment by exchange transfusion. Helv Paediatr Acta.

[10] Chan JCM, Goplerud JM, Papadopoulou ZL, Novello AC (1981). Kidney failure in childhood. Int J Pediatr Nephrol.

[11] Novak RW, Martin CR, Orsini EN (1983). Hemolytic-uremic syndrome and T-cryptantigen exposure by neuraminidase-producing pneumococci: an emerging problem?. Pediatr Pathol.

[12] Alon U, Adler SP, Chan JC (1984). Hemolytic-uremic syndrome associated with Streptococcus pneumoniae. Report of a case and review of the literature. Am J Dis Child.

[13] Feld LG, Springate JE, Darragh R, Fildes RD (1987). Pneumococcal pneumonia and hemolytic uremic syndrome. Pediatr Infect Dis J.

[14] Mizusawa Y, Pitcher LA, Burke JR, Falk MC, Mizushima W (1996). Survey of haemolytic-uraemic syndrome in Queensland 1979–1995. Med J Aust.

[15] Constantinescu AR, Bitzan M, Weiss LS, Christen E, Kaplan BS, Cnaan A, Trachtman H (2004). Non-enteropathic hemolytic uremic syndrome: causes and short-term course. Am J Kidney Dis.

[16] Cabrera GR, Fortenberry JD, Warshaw BL, Chambliss CR, Butler JC, Cooperstone BG (1998). Hemolytic uremic syndrome associated with invasive Streptococcus pneumoniae infection. Pediatrics.

[17] Kaplan SL, Mason EO, Basson WJ, Wald ER, Arditi M, Tan TQ, Schutze GE, Bradley JS, Givner LB, Kim KS, Yogev R (1998). Three year multicenter surveillance of systemic pneumococcal infections in children. Pediatrics.

[18] McTaggart SJ, Burke JR (1998). Streptococcus pneumoniae-induced haemolytic uraemic syndrome. J Paediatr Child Health.

[19] Krysan DJ, Flynn JT (2001). Renal transplantation after streptococcus pneumoniae-associated hemolytic uremic syndrome. Am J Kidney Dis.

[20] Myers KA, Marrie TJ (1993). Thrombotic microangiopathy associated with Streptococcus pneumoniae bacteremia: case report and review. Clin Infect Dis.

[21] von Eyben FE, Szpirt W (1985). Pneumococcal sepsis with hemolytic-uremic syndrome in the adult. Nephron.

[22] McGraw ME, Lendon M, Stevens RF, Postlethwaite RJ, Taylor CM (1989). Haemolytic uremic syndrome and the Thomsen Friendenreich antigen. Pediatr Nephrol.

[23] Martinot A, Hue V, Leclerc F, Chenaud M (1989). Haemolytic-uraemic syndrome associated with Streptococcus pneumoniae meningitis. Eur J Pediatr.

[24] Begue R, Dennehy PH, Peter G (1991). Hemolytic uremic syndrome associated with Streptococcus pneumoniae. N Engl J Med.

[25] Erickson LC, Smith WS, Biswas AK, Camarca MA, Waecker NJ (1994). Streptococcus pneumoniae-induced hemolytic uremic syndrome: a case for early diagnosis. Pediatr Nephrol.

[26] Pan CG, Leichter HE, Werlin SL (1995). Hepatocellular injury in Streptococcus pneumoniae-associated hemolytic uremic syndrome in children. Pediatr Nephrol.

[27] Sajjanhar T, Mayer A, Murdoch IA (1996). Chronic subdural haematoma and Streptococcus pneumoniae-associated haemolytic-uraemic syndrome. Acta Paediatr.

[28] Gilbert RD, Argent AC (1998). Streptococcus pneumoniae-associated hemolytic uremic syndrome. Pediatr Infect Dis J.

[29] Nathanson S, Deschenes G (2001). Prognosis of Streptococcus pneumoniae-induced hemolytic uremic syndrome. Pediatr Nephrol.

[30] Gatter N, Maier O, Hoppe B (2001). Streptococcus pneumoniae-induced hemolytic uremic syndrome: report of a further case. Pediatr Nephrol.

[31] Brandt J, Wong C, Mihm S, Roberts J, Smith J, Brewer E, Thiagarajan R, Warady B (2002). Invasive pneumococcal disease and hemolytic uremic syndrome. Pediatrics.

[32] Vanderkooi OG, Kellner JD, Wade AW, Jadavji T, Midgley JP, Louie T, Tyrell GJ (2003). Invasive Streptococcus pneumoniae infection causing hemolytic uremic syndrome in children: two recent cases. Can J Infect Dis.

[33] Cochran JB, Panzarino VM, Maes LY, Tecklenburg FW (2004). Pneumococcus-induced T-antigen activation in hemolytic uremic syndrome and anemia. Pediatr Nephrol.

[34] Proulx F, Sockett P (2005). Prospective surveillance of Canadian children with the haemolytic uraemic syndrome. Pediatr Nephrol.

[35] Barit G, Sakarcan A (2005). Antibiotic resistant Streptococcus pneumoniae and hemolytic uremic syndrome. Eur J Pediatr.

[36] Yong JH, Fonseca BK, Best EJ, Holland T, Craig ME (2005). A preventable illness? Purulent pericarditis due to Streptococcus pneumoniae complicated by haemolytic uraemic syndrome in an infant. Commun Dis Intell.

[37] Lee CF, Liu SC, Lue KH, Chen JP, Sheu JN (2006). Pneumococcal pneumonia with empyema and hemolytic uremic syndrome in children: report of three cases. J Microbiol Immunol Infect.

[38] Huang YH, Lin TY, Wong KS, Huang YC, Chiu CH, Lai SH, Hsia SH (2006). Hemolytic uremic syndrome associated with pneumococcal pneumonia in Taiwan. Eur J Pediatr.

[39] von Vigier RO, Seibel K, Bianchetti MG (1999). Positive Coombs test in pneumococcus-associated hemolytic uremic syndrome: a review of the literature. Nephron.

[40] Mulder AH, Gerlag PG, Verhoef LH, van den Wall Bake AW (2001). Hemolytic uremic syndrome after Capnocytophaga canimorsus (DF-2) septicemia. Clin Nephrol.

[41] Tobe TJ, Franssen CF, Zijlstra JG, de Jong PE, Stegeman CA (1999). Hemolytic uremic syndrome due to Capnocytophaga canimorsus bacteremia after a dog bite. Am J Kidney Dis.

[42] Watanabe T (2001). Hemolytic uremic syndrome associated with influenza A virus infection. Nephron.

[43] Siegler RL (1995). Hemolytic uremic syndrome in children. Curr Opin Pediatr.

[44] Siegler R, Oakes R (2005). Hemolytic uremic syndrome; pathogenesis, treatment, and outcome. Curr Opin Pediatr.

[45] Siegler RL, Pavia AT, Christofferson RD, Milligan MK (1994). A 20 year population based study of postdiarrheal hemolytic uremic syndrome in Utah. Pediatrics.

[46] Canadian Pediatric Surveillance Program 2002 Results (2003) Canadian Pediatric Society, Ottawa, pp 27–28

[47] Huang DTN, Chi H, Lee HC, Chiu NC, Huang FY (2006). T-antigen activation for prediction of pneumococcus-induced hemolytic uremic syndrome and hemolytic anemia. Pediatr Infect Dis J.

[48] von Vigier RO, Fossali E, Crosazzo L, Bianchetti MG (2005). Positive Coombs test in postpneumococcal hemolytic-uremic syndrome. Pediatr Infect Dis J.

[49] American Academy of Pediatrics (1997). Therapy for children for invasive pneumococcal infections. Pediatrics.

[50] Arditi M, Mason EO, Bradley JS, Tan TQ, Barson WJ, Schutze GE, Wald ER, Givner LB, Kim KS, Yogev R, Kaplan SL (1998). Three-year multicenter surveillance of pneumococcal meningitis in children: clinical characteristics, and outcome related to penicillin susceptibility and dexamethasone use. Pediatrics.

[51] Vierbuchen M, Klein PJ (1983). Histochemical demonstration of neuraminidase effects in pneumococcal meningitis. Lab Invest.

